# Reasons for low influenza vaccination coverage – a cross-sectional survey in Poland

**DOI:** 10.3325/cmj.2011.52.126

**Published:** 2011-04

**Authors:** Przemyslaw Kardas, Anna Zasowska, Joanna Dec, Magdalena Stachurska

**Affiliations:** First Department of Family Medicine, Medical University of Lodz, Lodz, Poland

## Abstract

**Aim:**

To assess the reasons for low influenza vaccination coverage in Poland, including knowledge of influenza and attitudes toward influenza vaccination.

**Methods:**

This was a cross-sectional, anonymous, self-administered survey in primary care patients in Lodzkie voivodship (central Poland). The study participants were adults who visited their primary care physicians for various reasons from January 1 to April 30, 2007.

**Results:**

Six hundred and forty participants completed the survey. In 12 months before the study, 20.8% participants had received influenza vaccination. The most common reasons listed by those who had not been vaccinated were good health (27.6%), lack of trust in vaccination effectiveness (16.8%), and the cost of vaccination (9.7%). The most common source of information about influenza vaccination were primary care physicians (46.6%). Despite reasonably good knowledge of influenza, as many as approximately 20% of participants could not point out any differences between influenza and other viral respiratory tract infections.

**Conclusions:**

The main reasons for low influenza vaccination coverage in Poland were patients’ misconceptions and the cost of vaccination. Therefore, free-of-charge vaccination and more effective informational campaigns are needed, with special focus on high-risk groups.

Due to natural variation, influenza incidence in Poland has dropped significantly over the last years in comparison with some years of the last decade of the 20th century. It is nevertheless still high: data from 2008 point to the incidence of approximately 600 per 100 000 population (1). The real incidence of the disease is underestimated, as the considerable portion of influenza cases and the resulting hospitalizations are still not registered (2). Moreover, characteristic for influenza is the occurrence of periodic pandemics. Thus, medical consequences of influenza in the country are serious, putting this infection in the focus of the public health measures.

An effective method of reducing influenza morbidity and mortality is vaccination. There are several inactivated influenza vaccines available in Poland, which were proven to prevent influenza infection and its complications in up to 70%-90% of vaccinated persons (2,3).

Since 1994, influenza vaccination has been recommended by national Protective Vaccination Program. Currently, it is recommended for healthy children (from 6 months to 18 years), elderly patients (55 and over), patients with chronic conditions (asthma, diabetes, heart failure, pulmonary dysfunction, and renal failure), and those with low immunity (4). However, vaccination is still not reimbursed from the national health insurance and the patients need to pay 100% of the price themselves. The average cost of the vaccine approximates € 8. Several initiatives undertaken by local authorities in the recent years enabled some high-risk patient groups provision of free of charge influenza vaccination (5). Unfortunately, these initiatives have been limited and have not resulted in significant improvements in influenza vaccination coverage.

Influenza virus tends to create new strains, characterized by diverse antigen structures. For this reason, the vaccine is modified yearly, according to the recommendations of the World Health Organization (6). Consequently, effective influenza protection is only achievable provided that the vaccination is repeated every year. This makes the influenza vaccination acceptance even more challenging. Recent reports suggest that the influenza vaccination coverage in Poland is far from satisfactory, actually one of the lowest in Europe (7,8). According to recent Vaccine European New Integrated Collaboration Effort Project publication, the number of influenza vaccine doses used in Poland during 2007-8 influenza season in high-risk groups (those aged over 65 and with underlying conditions) was lowest among the 15 studied European countries (9).

In order to help design future informational campaigns, the aim of this study was to assess the knowledge of influenza and attitudes toward influenza vaccination as factors related to low influenza vaccination coverage in Poland.

## Material and methods

This was a cross-sectional, anonymous, self-administered survey on a convenience sample (n = 640) of primary care patients aged 18 and over in Lodzkie voivodship (central Poland) in the period January 1 to April 30, 2007 (Table 1). The patients visited their primary care physicians for various reasons and as healthy companions of sick patients. The survey was based on a 28-item questionnaire, specially constructed for this purpose, including questions (in most cases open-ended) assessing patients’ knowledge of influenza (12 items, including 8 open-ended questions), attitudes toward influenza vaccination (5 items, including 2 open-ended questions), practice of influenza vaccination (4 items), and patients’ characteristics (7 items). The English translation of the questionnaire is available in the web-extra material 1.(web extra material 1)

**Table 1:** Comparison of sociodemographic characteristics between the study participants and general population of Poland

Characteristic	No. (%) of participants (n = 640)	General population (%)*
Age:		
range	20-90	
average (±standard deviation)	46.3 ± 16.6	
18-54 years	426 (66.6)	67.4
55 years and more	214 (33.4)	33.6
Sex:		
women	359 (56.1)	52.4
men	281 (43.9)	42.7
Occupation:		
blue-collar workers	60 (9.4)	44.7
white-collar workers	195 (30.5)	
students	71 (11.1)	6.1
farmers	5 (0.8)	6.8
unemployed	29 (4.5)	6.1
pensioners	123 (19.2)	30.2
other	45 (7.0)	
not provided	112 (17.5)	
Place of residence (population):		
<10 000	98 (15.3)	45.0
10 000-50 000	147 (23.0)	17.9
50 000-100 000	50 (7.8)	8.4
100 000-500 000	86 (13.4)	17.2
>500 000	258 (40.3)	11.5

*Data for general population of Poland (age 18 and older), according to the national Central Statistical Office (10).

The data were analyzed by descriptive statistics. Participants were divided into two groups: 18-54 years and 55 years and older, according to the definition of high-risk groups in the national recommendations on influenza vaccination (4). For the analysis of qualitative variables, χ^2^ test was used.

## Results

### Knowledge of influenza and influenza vaccination

The majority of participants (93.0%) believed that influenza was transmitted through the air and 25.9% believed that the infection could be caught by sharing kitchen utensils with the sick person (Table 2). Other ways of influenza spread were mentioned by fewer than 10% of participants.

**Table 2:** Knowledge of influenza and influenza vaccination among study participants from Poland (n = 640)

Item	Percentage
Most frequent ways of influenza virus transmission:*	
droplet infection	93.0
common cutlery and dishes	25.9
contaminated food products	8.6
contact with infected blood	6.1
breast-feeding	5.3
sexual contact	3.3
difficult to say	5.5
Initial symptoms of influenza:^†^	
fever	82.0
runny nose	39.8
muscle pain	39.1
headache	29.2
malaise	28.6
cough	26.3
bones and joints aches	21.1
chills	14.8
sore throat	7.7
gastrointestinal symptoms	2.3
In what sense is influenza different from the common cold?^†^	
higher, longer-lasting temperature	27.2
influenza leads to complications	23.1
Influenza has more severe symptoms	22.5
I do not know	17.8
muscle, bone, and joints aches	15.0
influenza is a viral disease	10.3
long-lasting malaise and weakness	5.8
cough and runny nose	4.5
there is no difference	3.1
headache	1.9
influenza should be treated with antibiotics	1.4
gastrointestinal symptoms	1.3
influenza is accompanied by airways inflammation	1.3
influenza should not be treated with antibiotics	1.3
influenza occurs in epidemics	0.5
Is influenza hazardous to one’s health?	
yes	91.1
no	2.2
difficult to say	6.6
Which influenza consequences might be hazardous for one’s life?^†^	
heart diseases	47.1
respiratory disease	27.8
inflammatory complications	15.3
I do not know	14.8
arthritis	10.3
meningitis	6.7
death	3.0
susceptibility to infections	2.5
otitis media	2.2
kidney disease	1.4
myositis	0.5
lymphadenopathy	0.3
chronic cough	0.3
Who should by all means receive influenza vaccine?^†^	
elderly	41.9
children	36.9
people with low immunity to viral infections	25.0
I do not know	14.4
health service workers	12.5
those in contact with large numbers of people	8.0
everyone	6.4
chronically ill	4.7
teachers	4.4
not everyone needs vaccination	2.5
farmers, people who have contact with animals	2.0
those who had influenza and are susceptible to disease	1.6
pregnant women and breastfeeding mothers	0.5
Who should not receive influenza vaccine?^†^	
healthy, immune people	12.7
chronically ill	11.1
there is no such group of people	8.9
people allergic to influenza vaccine	8.6
infants, and children	6.7
pregnant women and breastfeeding mothers	4.7
people with immune deficiency	4.4
people with medical contraindications	2.2
elderly	1.4
I do not know	42.5
When is the best time to get vaccinated against influenza?^†^	
autumn (September-November)	66.3
early autumn, before the influenza season	9.1
spring	7.5
winter (December-February)	3.9
summer	3.3
autumn-winter period	1.6
I do not know	14.7
Does influenza vaccine enhance the immunity against all types of viral infections?	
yes	19.5
no	47.0
difficult to say	33.4
Does influenza vaccine protect against the avian flu?	
yes	7.2
no	60.5
difficult to say	31.7
How often should the vaccination against influenza be carried out in order to guarantee the highest effectiveness?^†^	
1 time a year	63.4
2 times a year	6.6
at least 1 time a year, before each influenza season	3.1
every 2 years or less frequently	0.9
2 years +1 year break	0.2
I do not know	25.6

* Participants could choose more than one answer.

†Answers to the open question.

As symptoms characteristic for the initial stage of the infection, participants most often mentioned high temperature (82.0%), runny nose (39.8%), and muscle pain (39.1%). As characteristics differentiating influenza from the common cold, participants most often mentioned higher and longer-lasting temperature (27.2%), complications (23.1%), heavier course of the sickness (22.5%), as well as muscle, bone, and joint pains (15.0%). However, 17.8% of participants could not list any symptom differentiating the common cold from influenza and 3.1% of them believed that these conditions did not differ at all. The vast majority of participants (91.1%) were convinced that influenza infection could be dangerous for health; as its dangerous consequences, participants most often mentioned heart diseases (47.0%) and respiratory tract infections (27.8%).

When asked which groups of people “should by all means receive influenza vaccine,” participants in the first place mentioned elderly people (41.9%), children (36.9%), and people with low immunity to viral infections (25.0%). When asked who did not need to be vaccinated, participants mentioned healthy and immune people (12.7%), as well as the ill, including those with chronic diseases (11.1%). A considerable part of the participants did not know any contraindications for the vaccination (42.5%) and 4.7% mentioned pregnant and breastfeeding women as groups who should avoid vaccination.

According to 66.3% of the participants, it was best to receive vaccination in autumn. The majority of the participants (63.4%) were aware that influenza vaccination should be repeated every year. However, every fourth participant (25.6%) did not have an opinion on that subject. Almost 20% of the participants believed that influenza vaccination improved immunity to all viral infections. The belief that influenza vaccination protects against the avian influenza was shared by 7.2% of the participants. No major differences in patients’ knowledge of influenza were observed depending on age and sex.

### Practice of influenza vaccination

There were 20.8% of the participants who had been vaccinated against influenza in the 12 months before the survey. This percentage was similar for both sexes (20.7% of men and 20.6% of women, *P* > 0.05). It was only slightly higher for older participants (19.2% in 18-54 age group and 23.8% in ≥55 age group, *P* > 0.05). Altogether 29.7% of participants had received vaccination in the previous years, 60.0% of whom had also received it in the 12 months before the survey. Vaccination was most often done in the primary health settings, work place, and patients’ homes (54.1%, 24.1%, and 18.0% of the vaccination cases, respectively). Before vaccination, fewer than three fourths of vaccinated participants were examined by a physician (74.4%). After vaccination, various adverse effects occurred in 60.2% individuals, most often local pain in the place of injection (34.6%), general bad feeling (24.1%), and muscle pains (12.0%). Majority of the vaccinated participants thought that vaccination had a positive effect on them (65.4%), although some had a contrary opinion (3.8%).

The majority of participants who had not had influenza vaccination in the 12 months before the study, explained it with good health, lack of belief in the vaccine effectiveness, and the high cost of the vaccine (Table 3). Women listed good health as the reason more often than men (33.1% vs 22.2%, *P* < 0.01), whereas men listed lack of belief in vaccine effectiveness more often than women (22.2% vs 13.4%, *P* < 0.01). Younger patients (18-54 years) listed good health as the reason considerably more often than older ones (31.4% vs 18.4%, *P* < 0.01).

**Table 3:** Reasons for not taking the influenza vaccination among study participants from Poland*

	Percent of participants in age group		
Reason for not taking influenza vaccination	18-54 years (n = 344)	55 years and more (n = 163)	total (n = 507)
Good health	31.4^†^	18.4^†^	27.2
Lack of belief in vaccination effectiveness	14.8	20.9	16.8
High cost of vaccination	9.3	10.4	9.7
Lack of time	10.8	5.5	9.1
Used to treat influenza with natural remedies	3.5	6.7	4.5
Due to the health state (chronically sick, was sick in the vaccination period)	2.9	2.5	2.8
Did not have an opportunity to receive vaccination	1.5	3.1	2.0
Had vaccination in previous years, and thinks that it is not necessary to repeat it	2.0	1.8	2.0
Vaccination reduces immunity; once vaccinated, one have to repeat it every year	2.3	0.6	1.8
Difficult to say	21.2	28.8	23.7

*For the open question “Why have you not taken a vaccine against influenza?”; patients were allowed to give more than one answer. This question was only answered by those who had not received vaccination within 12 mo before the study, hence the number is lower than the total number of participants (507 vs 640).

†χ^2^ test, *P* < 0.01.

**Figure 1:**
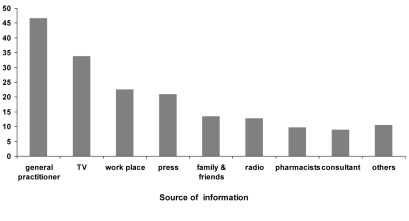
Sources of participants’ knowledge on the possibility of influenza vaccination, as answers to a closed question. Percentages do not sum up to 100% since participants were allowed to give more than one answer.

The participants most often learnt about the possibility of influenza vaccination from their family physicians, television, and at work place (Figure 1).

## Discussion

Our study found that low influenza vaccination coverage in Poland was related to patients’ false beliefs and high cost of influenza vaccination.

Influenza vaccination is a safe, effective, and cost-effective method of preventing influenza infections (11). However, the rate of influenza vaccination use in Poland is still unsatisfactory (7). Although it was growing systematically till 2004 (data from the years 1992-2002 indicate the growth of over 200 times, up to more than 100 doses per 1000 of population) (12), in the following years the trend was reversed, despite lack of problems with vaccination supply. In the epidemiological season 2007/08, only 9.5% of the Polish general population received the vaccine (13). Among persons aged 65 and over, the vaccine coverage increased only from 7% in 2004 to 14% in 2007 (7). The coverage of 13.9% of elderly people in Poland in the 2007/2008 season is the lowest of the 11 studied countries (13). A survey conducted in university hospitals found that only 22.3% of physicians, 10.6% of nurses, and 13.4% of medical students regularly took vaccines against influenza (14). Vaccination coverage among health care workers in Poland in the 2007/2008 season was 6.4%, the lowest among the 11 European countries studied (13).

These vaccination rates place Poland far behind other European countries, where vaccination coverage reaches 25% (15,16), or the USA, where in 2004/2005 season 42% of adults from high-risk groups received vaccination, as well as 62.7% of people aged over 65 years (17). The WHO encouraged member countries to reach the vaccination level of 75% among high-risk patient groups by the year 2010, as did the European Parliament. Recently, the Council of European Union has recommended reaching 75% vaccination coverage in older-age groups as early as possible, and preferably by the 2014-2015 winter season (18). Therefore, the need of popularizing influenza vaccination is of the uttermost importance in the Polish society, particularly in high-risk groups, such as the elderly and chronically ill, and among health care workers.

Participants in this study showed a considerable knowledge of influenza, its symptoms, ways of transmission, and complications. However, several illogicalities and misconceptions are worth pointing out. Over 90% of participants were convinced that influenza can be dangerous for health but only 42% of them believed that that the elderly were a priority population for vaccination. For 11% of participants, chronic conditions seemed to be contraindications to vaccination. Moreover, a large portion of participants could not point out any difference between influenza and other viral respiratory tract infections (common colds). Listing runny nose as a typical influenza symptom and believing that influenza vaccination was effective against all types of viral infections (almost 20% of participants) were also common misconceptions. These misconceptions are important, as in many cases, they may lower the motivation for the vaccination. This is especially important for those who had taken the vaccination before. For them, catching a different type of viral disease may prove the influenza vaccination to be ineffective. According to our study, only 60% of previously vaccinated participants had been revaccinated in the year before the survey. Therefore, there is a need for campaigns designed to increase the influenza vaccination rate in Poland that should clearly inform on the scope of influenza vaccination effectiveness and the differences between influenza and other viral respiratory tract infections.

Recent outbreak of the A(H1N1) 2009 virus (“swine flu”) and the pandemic announced by the WHO experts contributed greatly to this problem (19). Poland was the only EU country that refused to order pandemic vaccine for its citizens (20). The Ministry of Health announced an official statement in the Polish Parliament in which it not only underestimated the risk of the swine flu, but also questioned the effectiveness and safety of the pandemic vaccine (21). The Poles believed that message and it went in parallel with their reluctance toward vaccination: according to the Eurobarometer study, as many as 45% of the Poles believed that the vaccination against swine flu could not protect them from the infection (22). However, it seems that people extended that message to the regular influenza vaccination as well. Recent reports from infective season 2009/2010 prove that this “negative promotion” of influenza vaccination in the mass media was very effective: the coverage rates dropped to 6.5% of the general population (23).

The most frequently mentioned reason for not taking influenza vaccination was good health. However, it is noteworthy that nearly 10% of the participants mentioned financial aspects. Similar observations were made in other studies in Poland. Kroneman et al showed that in Poland in 2004 and 2005, 34% and 36% of participants from high-risk groups, respectively, had misconceptions preventing them from receiving vaccination (ie, they believed they were “resistant to influenza”), while 24% of the participants in both years stated that they would not have a vaccination due to financial difficulties (24). Kalinowski et al found that 37% of students did not receive vaccination due to belief in their good health and 6% due to the cost of the vaccine (25).

Some authors outside of Poland believe that the lack of improvement in influenza vaccination coverage in Poland over the last few years and a constant level of patients’ false beliefs point to a lack of effective national strategy for influenza vaccination promotion (26). The strategy should embrace free-of-charge provision of influenza vaccination, giving priority to the high-risk groups, especially the elderly (27). The need to pay for the vaccination out of pocket is a strong discouraging factor. In the USA, it was found that introducing a fee of US $10 for a vaccine would decrease the number of vaccinated people by 1/3 (28). At the same time, there is evidence that reimbursing costs of influenza vaccination increases vaccination coverage (29).

High percentage of people who made a conscious decision not to receive influenza vaccination in this survey and a lack of belief in the effectiveness of vaccination (16.8%) indicate that benefits of influenza vaccination should be demonstrated to the public, particularly to the high-risk groups.

This survey proved that primary health settings were not only the most frequent setting where influenza vaccination takes place, but also the most frequent source of information about the need for vaccination. This does not mean, however, that further action promoting vaccination against influenza should not be conducted at this level. The experience from other countries teaches us that family physicians are those who are most capable of persuading patients to receive influenza vaccination (16), and interventions taken at this level, including both physicians and patients, contribute to popularization of influenza vaccination (30,31). Personalized invitations to vaccination at family physicians’ practices are worth applying in the national program of influenza vaccination, as they were found to be particularly effective (27). The media, on the other hand, played hardly any role in the promotion of influenza vaccination in Poland (25). We hope that such situation will soon change.

The obvious limitation of this study is the method used for participants’ enrolment. Although the study was performed in a number of primary health care practices in Lodzkie voivodship and the study sample corresponds well with age and sex distribution of the Polish society (Table 1), the convenience sample, with overrepresentation of large-city dwellers, did not guarantee the full representativeness of the study for the Polish population. Moreover, the survey covered the people who visited their primary care physician. Thus, it could be biased toward people with greater interest in one’s health in general and influenza vaccination in particular. We could not perform further analysis of participants’ characteristics except sex and age because almost a quarter of them did not state their occupation or stated “other” in the questionnaire. Other characteristics were not collected.

Since major reasons for low influenza vaccination coverage in Poland include patients’ false beliefs and high cost of vaccination, it is necessary to organize informational campaigns that point out the differences between influenza and other viral respiratory tract infections, as well as to make the vaccination available free of charge, especially for high-risk groups.
