# Supplementary material for: Reasons for low influenza vaccination coverage – a cross-sectional survey in Poland
Source: Croat Med J. 2011 Apr;52(2):126–33. doi: 10.3325/cmj.2011.52.126 (PMC3081210; doi:10.3325/cmj.2011.52.126)
Supplement: Supplementary Table 1 [file CroatMedJ_52_s001.pdf]

Appendix I – survey questionnaire

Survey on influenza vaccination

Dear Madam, Sir!

We would like you to complete a short survey on the flu. Information gathered in this survey will be used for scientific research conducted by the First Department of Family Medicine, Medical University of Lodz. This survey is of course completely voluntary, and completely anonymous.

Thank you for completing the survey!

1. **Please specify how it comes to influenza virus infection (You can select more than one answer)**

1☐ droplet way
2☐ consumption of contaminated food products
3☐ contact with infected blood
4☐ by breast-feeding
5☐ using common cutlery and dishes
6☐ through sexual contact
7☐ difficult to say

2. **Please specify what the first symptoms of flu are**

- ....
- ....
- ....

3. **In what sense is the flu different from the common cold?**

- ....
- ....
- ....

4. **May the flu be hazardous for one's health?**

1☐ Yes
2☐ No **-> go to q. 6**
3☐ difficult to say **-> go to q. 6**

5. **What kind of influenza consequences might be hazardous for one's life?**

....
……..

6. **Have you received the vaccination against influenza within last 12 months?**

1☐ Yes **-> go to q. 8**
2☐ No

7. If no, why??

....

....**-> go to q. 14**

8. If yes, why?

....

....

9. Were you examined by a physician prior to the vaccination?

1☐ Yes

2☐ No

10. Where did this vaccination take place?

1☐ GP / in primary care

2☐ at home

3☐ in the workplace

4☐ in another place - give details...

11. How did you find out about the possibility of vaccination?

(You can select more than one answer)

1☐ from family doctor / primary care

2☐ from a specialist

3☐ from the pharmacist

4☐ from TV

5☐ from radio

6☐ from the press

7☐ from the employer

8☐ from friends

9☐ other - give details..

12. Did any worrying symptoms occur after the vaccination?

(You can select more than one answer)

1☐ soreness at the injection site

2☐ fever

3☐ malaise

4☐ muscle pain

5☐ headache

6☐ allergic reactions – give details ?... ....

7☐ other - give details..

13. Do you think that the vaccination had an impact on your health?

1☐ yes, positive

2☐ yes, negative

3☐ I did not notice any change

14. Have you taken influenza vaccination in the past (not earlier than 12 months ago)?

1☐ Yes

2☐ No

15. Who should by all means receive influenza vaccine?

- ....
- ....

16. Who should not receive it?

- ....

17. **When is the best time to get vaccinated against influenza? (Please give the name of the month or season)**

....

18. Do you think that the flu vaccination enhances the immunity against all types of viral infections?

1☐ Yes

2☐ No

3☐ Difficult to say

19. Does the flu vaccine protect against the so-called "avian flu"?

1☐ Yes

2☐ No

3☐ Difficult to say

20. Are there any other methods for preventing influenza, besides vaccination?

....

....

21. How often should the vaccination against influenza be carried out in order to guarantee the maximum effectiveness?

....

22. **Do you suffer from any of the chronic conditions listed below? (You can select more than one answer).**

1☐ hypertension

2☐ coronary artery disease

3☐ diabetes

4☐ asthma, chronic bronchitis, COPD
5☐ cancer
6☐ kidney diseases
7☐ other - please specify..

23. **Are you taking any medications regularly? If so, please specify.**

....

……………………………………………………………………………………..

24. Have you been hospitalised within the last 12 months?

1☐ Yes - for what reason? ... ...
2☐ No

Your data:

25. Age ....years

26. Gender:

1☐ female
2☐ male

27. Occupation: ………………………..…………

28. What is the population of your place of living?

1☐ Below 10 000
2☐10 000 -50 000
3☐ 50 000-100 000
4☐ 100 000 -500 000
5☐ More than 500 000

Thank you.
